# Therapeutic effect of histone deacetylase 6 inhibitor for a mouse model of phenylketonuria

**DOI:** 10.1038/s41598-025-29143-7

**Published:** 2025-11-22

**Authors:** Jung Eun Choi, Saeyoung Park, Hyeryung Song, Il Tae Hwang, Sung-Chul Jung, Hae Soon Kim

**Affiliations:** 1 Department of Pediatrics, Ewha Womans University School of Medicine, 260, Gonghang-daero, Gangseo-gu, 07804 Seoul, Republic of Korea; 2 Department of Biochemistry, Ewha Womans University School of Medicine, 260, Gonghang-daero, Gangseo-gu, 07804 Seoul, Republic of Korea; 3 Department of Pediatrics, Kangdong Sacred Heart Hospital, Hallym University College of Medicine, Seoul, Republic of Korea

**Keywords:** Biochemistry, Genetics, Diseases, Endocrinology, Medical research, Molecular medicine

## Abstract

Phenylketonuria (PKU) is an inherited metabolic disorder caused by mutations in the phenylalanine hydroxylase (*PAH*) gene, leading to phenylalanine accumulation and clinical symptoms such as intellectual disability and hypopigmentation. Histone deacetylase 6 inhibitors (HDAC6i), known to promote the degradation of misfolded proteins, have shown potential in treating protein misfolding-related disorders. In this study, we evaluated the therapeutic effect of Tubastatin A, a selective HDAC6i, in *Pah*^*eun2*^ PKU model mice carrying a missense PAH mutation. Mice were treated with Tubastatin A for 12 weeks, and outcomes were assessed via phenylalanine levels, fur pigmentation, and expression of heat shock protein 90 (HSP90). Treated mice exhibited approximately a 50% reduction in plasma phenylalanine levels, partial recovery of black fur pigmentation, and increased expression of acetylated HSP90 in the liver. These findings suggest that HDAC6 inhibition may alleviate PKU symptoms by increasing HSP90 acetylation, which interferes with the degradation of misfolded PAH and may allow residual enzymatic activity. Our results support the therapeutic potential of HDAC6i in PKU and warrant further studies to optimize dosage and evaluate the long-term effects and safety of sustained treatment.

**Supplementary Information:**

The online version contains supplementary material available at 10.1038/s41598-025-29143-7.

## Introduction

Phenylketonuria (PKU) is an autosomal recessive inherited metabolic disorder caused by mutations in the phenylalanine hydroxylase (*PAH*) gene, leading to PAH enzyme deficiency. The loss of PAH enzyme activity impairs the catabolic pathway that converts phenylalanine to tyrosine; this reaction requires tetrahydrobiopterin (BH_4_) as an essential cofactor^1^. Elevated phenylalanine levels are neurotoxic and, if left untreated, can result in significant neurological damage including cognitive impairment, seizures, and behavioral abnormalities. Additional clinical features include hypopigmentation of the skin, hair, and eyes and growth failure^2–4^. Since the 1960s, neonatal screening for PKU has allowed early diagnosis and intervention, reducing the risk of severe intellectual disability^5^.

Treatment involves a lifelong low-phenylalanine diet to maintain low levels of phenylalanine^6^. This dietary treatment is most effective when initiated in early life. However, maintaining strict dietary adherence into adulthood remains challenging. Recent evidence suggests that reinitiating therapy in adulthood, including with pharmacological agents such as pegvaliase or sepiapterin, can improve neurocognitive and psychiatric symptoms in many patients^7^. Moreover, strict dietary therapy commonly leads to nutritional deficiencies in patients with PKU, such as vitamin B_12_, vitamin D, and calcium deficiencies. Recent studies also indicate that, even with early and strict dietary management, individuals with PKU tend to have lower IQ scores and psychosocial impairments compared to the general population^2,8^. Elevated blood phenylalanine levels are known to disrupt the synthesis of key neurotransmitters in the brain, particularly dopamine and serotonin, which may explain neuropsychiatric symptoms such as attention deficits, anxiety, and depression frequently observed in adults with poorly controlled PKU^7^.

Many studies have been performed to develop methods to efficiently remove excess phenylalanine, leading to various treatments that support or replace dietary restriction. Cofactor therapy using sapropterin (a synthetic BH_4_ analog) aids in reducing phenylalanine levels by activating residual PAH enzyme activity but is only effective in a subset of patients with BH4-responsive form of PKU​ with BH_4_ deficiency. For 90% of patients with classic PKU, BH_4_ therapy has no beneficial effects^5,9^.

The pathogenesis of PKU is primarily driven by missense mutations that reduce the stability and folding efficiency of the PAH enzyme, resulting in decreased activity^10^. Enzyme replacement therapy with pegvaliase, a pegylated form of phenylalanine ammonia lyase (PAL), offers an alternative approach by degrading phenylalanine directly in the bloodstream, bypassing the need for hepatic PAH activity. However, its necessity for daily subcutaneous injections and its immune response risks should be brings limitations for its long-term application​^11^. Gene therapy and mRNA therapy are also being investigated for their potential to correct PAH deficiency, though their cost and the need for long-term studies to verify safety remain significant challenges​^12–15^.

Histone deacetylase 6 (HDAC6) is a Class II HDAC primarily localized in the cytoplasm, where it plays key roles in deacetylating nonhistone proteins such as HSP90 and α-tubulin, and in promoting the degradation of misfolded proteins^16,17^​. Inhibiting HDAC6 may delay the rapid degradation of misfolded proteins, thereby allowing time for proper folding and partial restoration of function in mutant misfolded PAH proteins offering a novel pathway to enhance the stability of mutant misfolded proteins. Tubastatin A (TSA), a selective inhibitor of HDAC6 is being investigated as a potential treatment of various neurodegenerative diseases due to its ability to inhibit the degradation of misfolded proteins^18,19^.

In this study, we investigated the therapeutic effect of TSA, selective HDAC6 inhibitor, in *Pah*^*eun2*^ mice carrying a PAH missense mutation. We evaluated whether HDAC6 inhibition could reduce phenylalanine levels and improve phenotypic features, thereby offering a novel therapeutic strategy for PKU.

## Result

### Reduction of plasma phenylalanine levels TSA treatment in a PKU mouse model

**Fig. 1:**
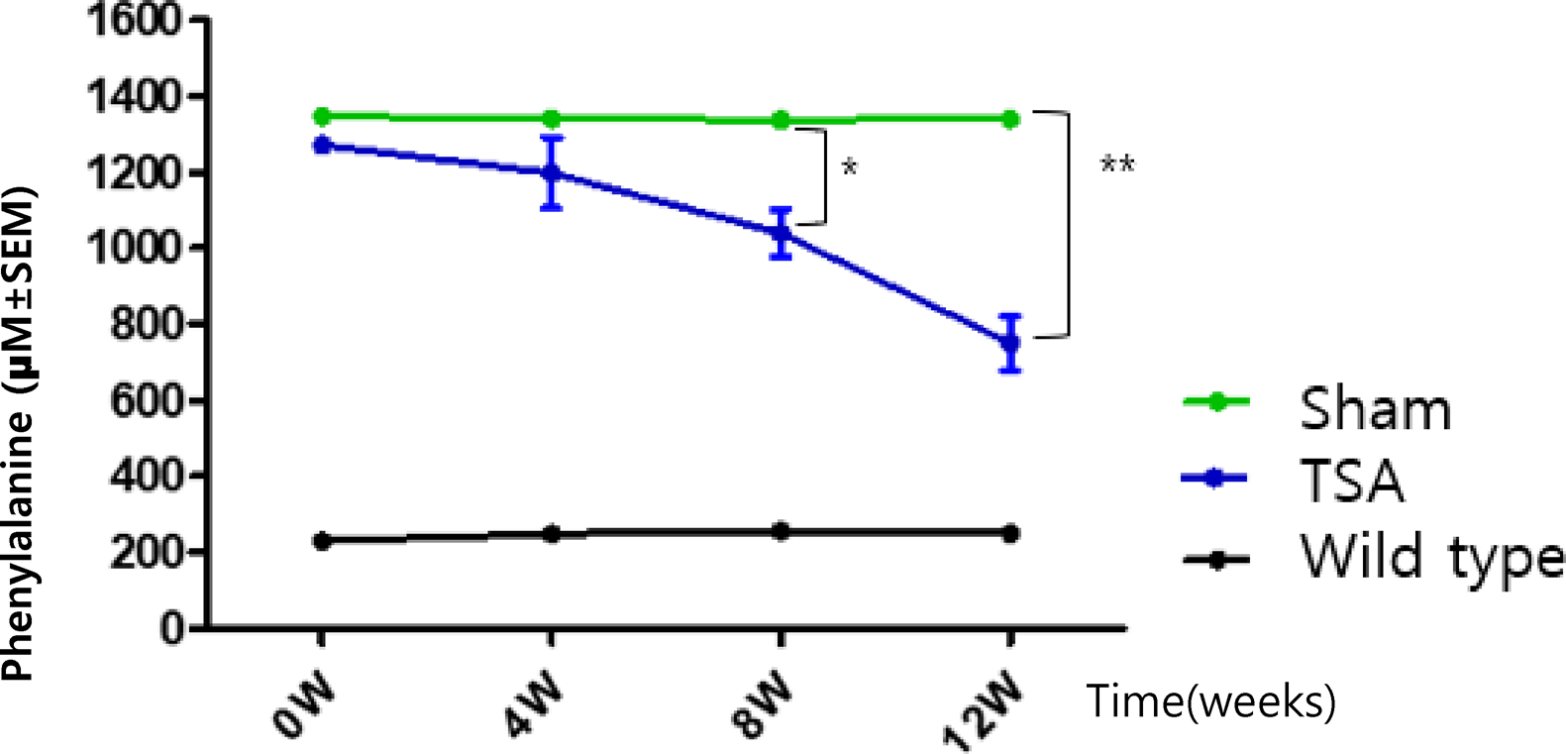
Changes in plasma phenylalanine concentration in *Pah*^*eun2*^ Mice over time.

The effects of TSA on phenylalanine level were observed in *Pah*^*eun2*^ mice following 12 weeks of twice-weekly intraperitoneal injections. TSA was dissolved and diluted in DMSO for administration, and to assess whether the dimethyl sulfoxide (DMSO) solvent had any effects, a sham group receiving DMSO alone was included under the same injection conditions. The plasma phenylalanine concentration in *Pah*^*eun2*^ mice was significantly reduced following 12 weeks of treatment with TSA (50 mg/kg), compared to the untreated (sham) group. The wild-type control group showed consistently low phenylalanine levels throughout the experiment, ranging from 230.9 to 289.6 µM (Table 1). The TSA-treated group showed a statistically significant reduction in plasma phenylalanine levels, decreasing to 1039.39 ± 36.27 µM at weeks 8 which was significantly lower than the sham group (1338.59 ± 8.76 µM, *p* < 0.05), and further reducing to 749.41 ± 42.16 µM at week 12, compared to the sham group (1341.59 ± 5.24 µM, *p* < 0.01) (Table 1; Fig. 1). These findings demonstrate the efficacy of TSA, an HDAC6 inhibitor, in reducing plasma phenylalanine levels in the *Pah*^*enu2*^ mouse model of PKU. The progressive reduction in plasma phenylalanine levels over the 12-week treatment period, approximately 50% compared to the sham group, highlights the therapeutic potential of managing hyperphenylalaninemia, thereby ameliorating PKU symptoms.

**Table 1:** Serum phenylalanine concentration in *Pah*^*eun2*^ mice and wile-type mice during 12-week TSA treatment.

Group		Phenylalanine (µM)			
		week 0	week 4	week 8	week 12
Sham (Untreated)	1	1349.1	1325.4	1329.8	1335.8
	2	1345.1	1355.1	1347.4	1346.2
	Mean ± SEM	1347.00 ± 2.00	1340.26 ± 14.89	1338.59 ± 8.76	1341.01 ± 5.24
TSA (50 mg/kg) (Treated)	3	1296.1	1241.2	981.4	672.1
	4	1257.7	1091	1030.7	758.8
	5	1340	1272.4	1068.1	866.9
	Mean ± SEM	1298.00 ± 23.80	1198.11 ± 53.89	1039.39 ± 36.27	749.41 ± 42.16
Wild type (Control)	6	230.9	246.5	241.3	242.8
	7	225.7	230.9	266.5	289.6
	8	233.8	270.3	260.6	239.0
	Mean ± SEM	253.30 ± 6.10	249.21 ± 11.45	256.14 ± 7.63	250.27 ± 9.43

Statistical significance between groups was analyzed using one-way ANOVA. *P < 0.05, **P < 0.01 indicate significant differences.

## Restoration of fur color in a PKU mouse model after TSA treatment

**Fig. 2:**
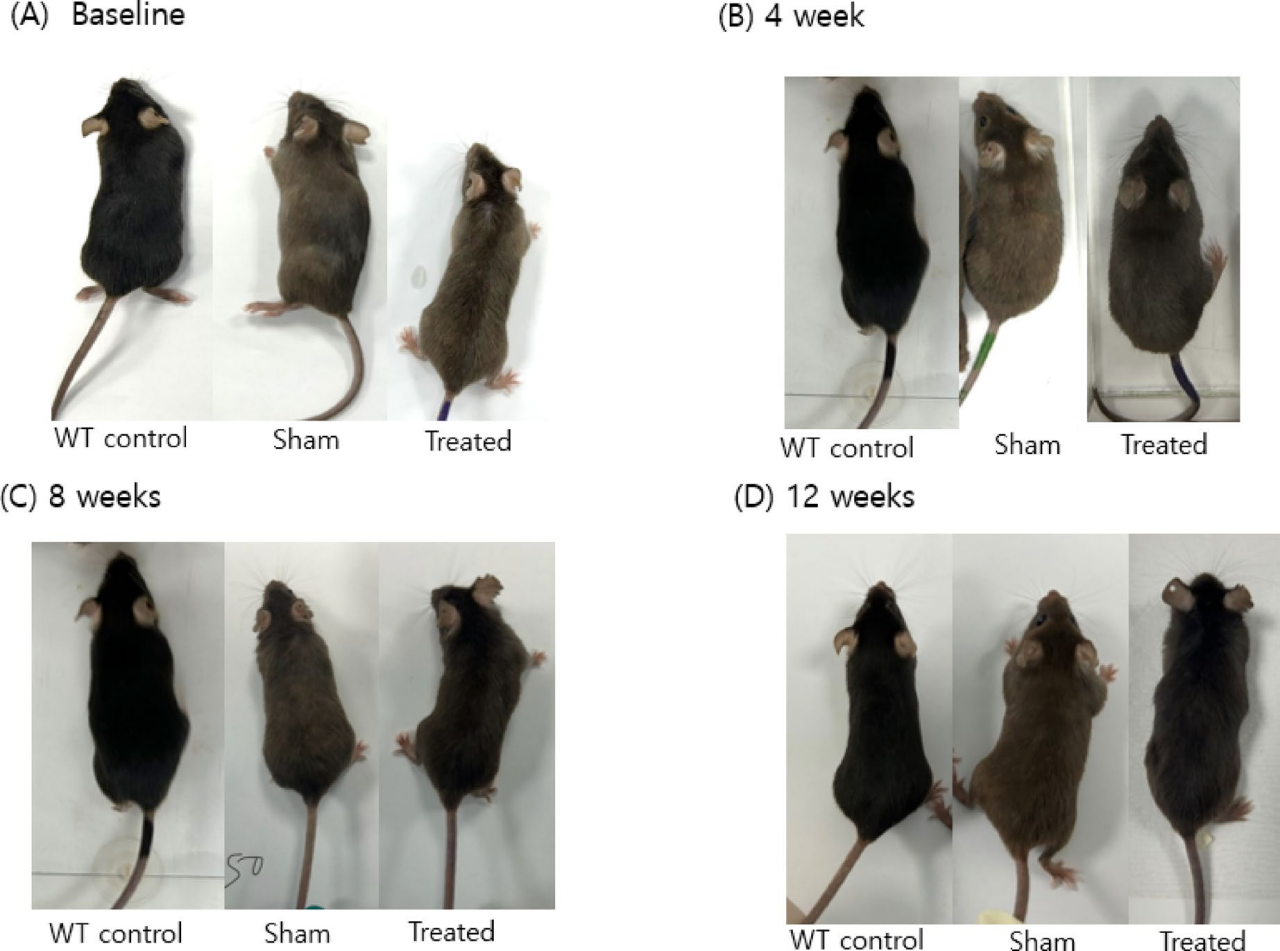
Coat color change among three groups of mice during 12-week experiment. (**A**) Before treatment (0 weeks), the fur of sham and treated Paheun2 mice is gray due to impaired phenylalanine metabolism, while the wild-type mice display black fur. (**B-D**) Progressive fur darkening is observed in the treated group beginning at 4 weeks, shows significantly improving by 8 weeks, and becoming nearly indistinguishable from WT mice by 12 weeks.

At baseline the fur of *Pah*^*eun2*^ mice in both sham and treated groups was gray, indicating the impaired phenylalanine metabolism function. Twelve weeks after treatment, a change in fur color to black was observed in *Pah*^*eun2*^ mice treated with TSA (50 mg/kg). Restoration of fur color began by week 4, progressed significantly by week 8, and by the end of the 12-week treatment period, the fur color of the treated *Pah*^*eun2*^ mice had almost completely transitioned to black (Fig. 2). The coat color change in the PKU mouse model reflects a recovery in the metabolic pathway converting phenylalanine to melanin, which depends on the proper function of the PAH enzyme. This phenotypic shift suggests improved PAH activity and reduced plasma phenylalanine levels. High-dose TSA (100 mg/kg) was initially tested; however, *Pah*^*eun2*^ mice in this group developed abdominal edema and died within eight weeks. Necropsy revealed kidney enlargement and ascites, indicating dose-dependent toxicity. In contrast, mice in wild-type control group displayed normal black fur throughout the experiment.

### Increase in PAH expression levels in the liver of the PKU mouse model after TSA treatment

**Fig. 3:**
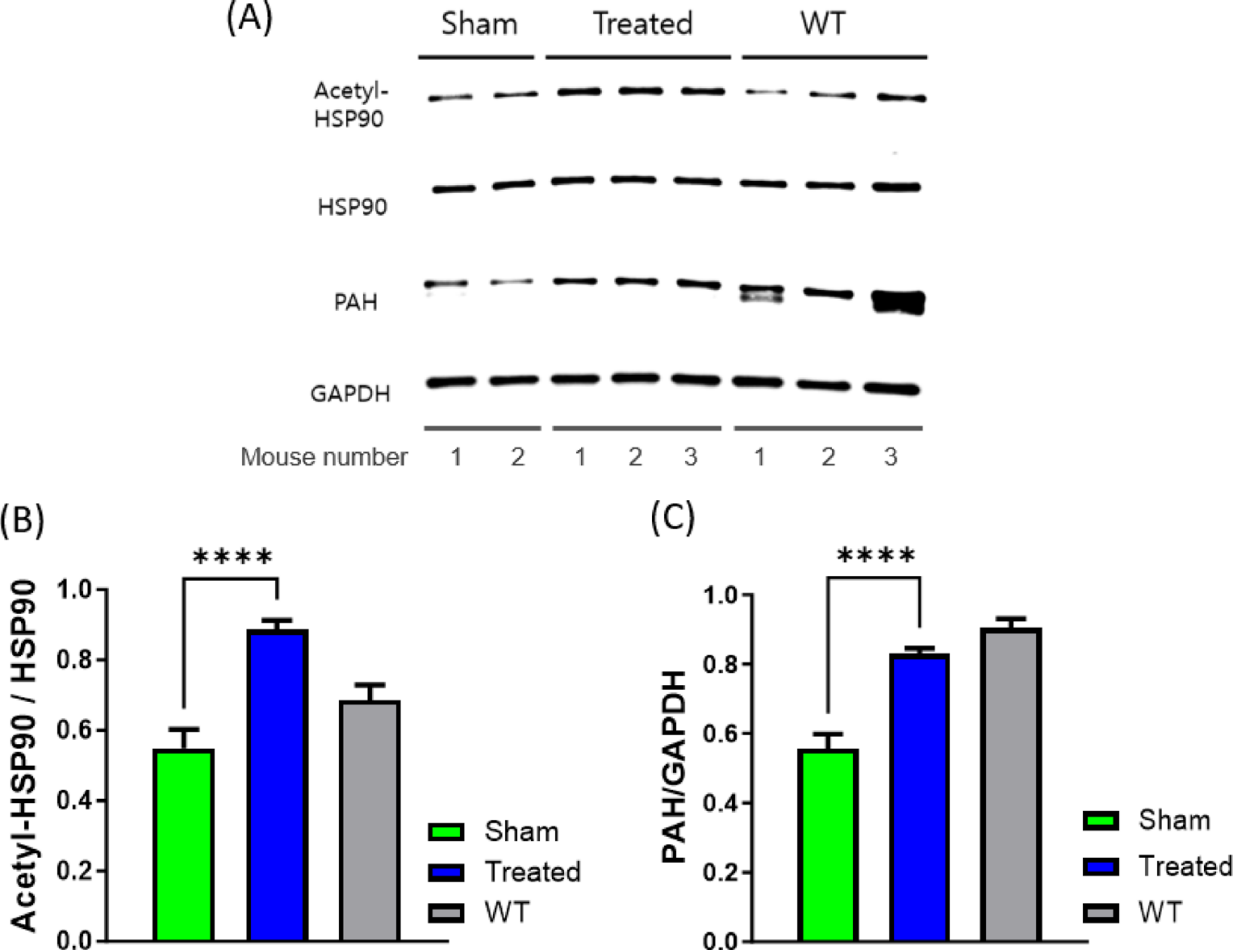
Western blot analysis of acetyl-HSP90, HSP90, and PAH protein expression in liver tissues from sham, treated, and wild-type groups following 12 weeks of TSA treatment. (**A**) Expression of acetyl-HSP90, HSP90, PAH, and GAPDH (used as a loading control). (**B**) Quantitative analysis of acetyl-HSP90 relative to HSP90 protein levels. (**C**) Quantitative analysis of PAH relative to GAPDH protein levels. The expression of PAE was quantified using the Image J software (Version 1.49) and expressed as arbitrary units (a.u.). Results are presented as mean ± SEM, with statistical significance indicated. ****P<0.0001.

To further evaluate the efficacy of TSA in improving PAH function, we conducted western blot analysis to assess PAH protein expression in the liver. We observed an increase in PAH protein expression in the liver of *Pah*^*eun2*^ mice treated with TSA compared with those in the sham group. PAH expression, which can serve as a diagnostic marker for PKU, was quantitively significantly higher in the treated group (0.83 ± 0.02 a.u.) than in the sham group (0.56 ± 0.04 a.u.) (Fig. 3). These findings indicate that HDAC6 inhibition enhances PAH protein expression by inhibiting the degradation of PAH, thereby contributing to increased PAH levels in the liver and ameliorating PKU symptoms in the animal model.

### Enhanced acetylation and expression of a key protein in the unfolded protein response, HSP90, after TSA treatment

**Fig. 4:**
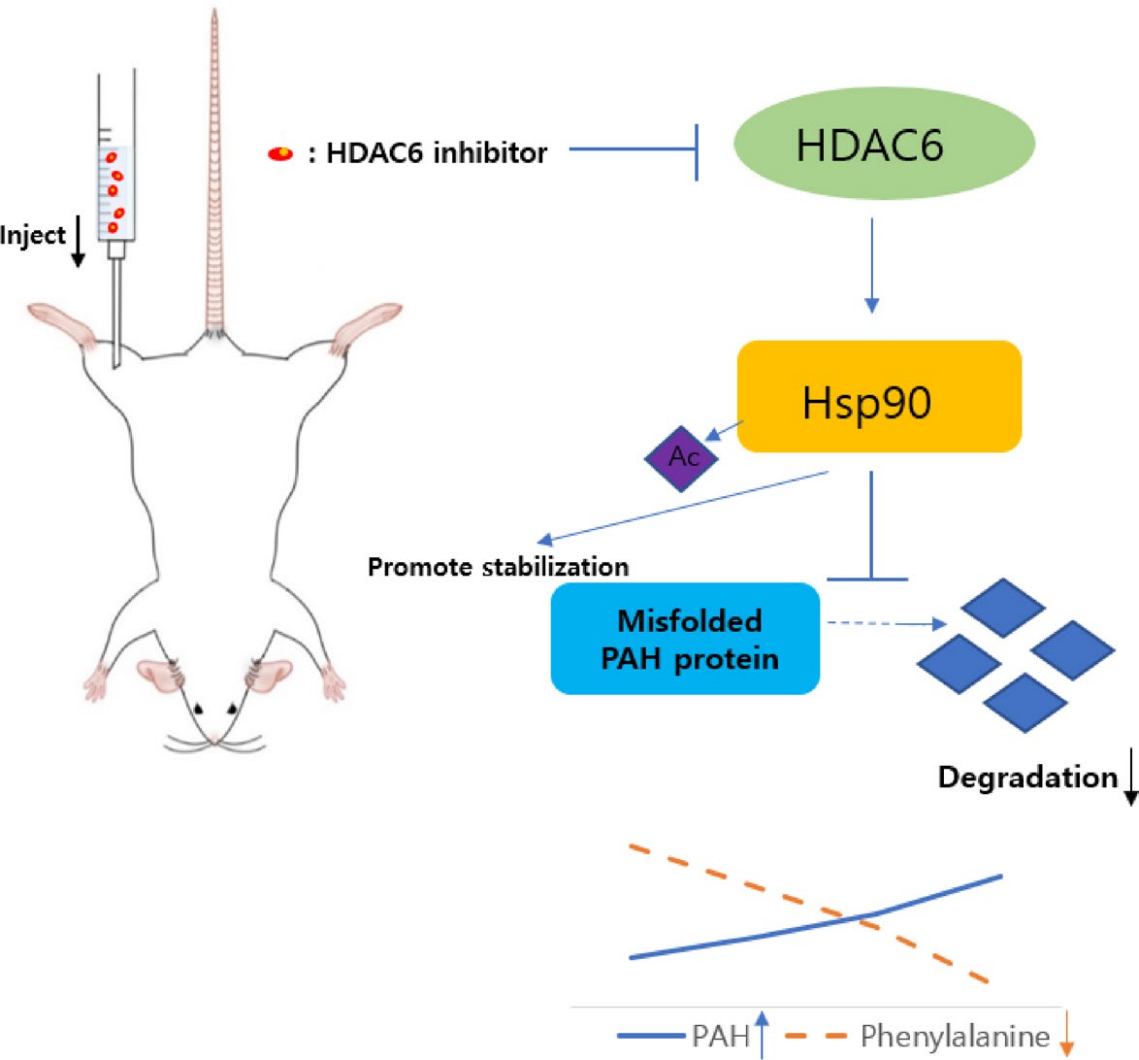
Mechanism of TSA in enhancing HSP90 acetylation and stabilizing misfolded PAH protein to improve phenylalanine metabolism.

In the western blot analysis, acetylated HSP90 was significantly increased in *Pah*^*eun2*^ mice treated with TSA, correlating with increased expression of the PAH protein in the liver (Fig. 3). TSA was shown to effectively enhance the stabilization of mutant PAH. HSP90, a molecular chaperone critical for the folding and degradation of client proteins, is deacetylated by HDAC6 under normal conditions^20^. HDAC6 plays a central role in regulating nonhistone protein acetylation such as ɑ-tubulin and HSP 90, which affects protein stability, degradation, and enzymatic activity. By inhibiting HDAC6, TSA prevents the deacetylation of HSP90, promoting its acetylated form. Acetylated HSP90 is associated with improved stabilization of ɑ-tubulin microtubules and client proteins, while impairing chaperone function of HSP90, thereby inhibiting degradation of misfolded protein through natural degradation pathways such as mutant PAH enzyme. Therefore, partially active residual misfolded PAH enhances phenylalanine metabolism, thereby reducing phenylalanine levels and alleviating symptoms (Fig. 4).

## Discussion

This study identified the therapeutic potential of TSA, a selective HDAC6 inhibitor, in a PKU mouse model carrying a missense mutation in the PAH gene, resulting in a reduction in plasma phenylalanine levels. Plasma phenylalanine levels in TSA-treated *Pah*^*eun2*^ mice progressively decreased over the 12-week treatment period, with significant reductions observed at week 8 and further reductions at week 12 compared to the sham group. Moreover, treated mice showed a phenotypic restoration of fur color from gray to black, beginning at week 4 and nearly complete by week 12. These biochemical and phenotypic changes, critical criteria for evaluating the amelioration of PKU symptoms, indicate the therapeutic potential of TSA. Western blot analysis of liver tissue collected after 12 weeks of treatment revealed increased acetylated HSP90, a substrate of HDAC6, and PAH protein expression in TSA-treated mice. These findings suggest that the HDAC6i such as TSA prevents the degradation of misfolded PAH proteins and restore partial enzymatic activity by enhancing HSP90 acetylation.

A previous study had revealed that HDAC6 plays a critical role in regulating protein aggregation, axonal transport, and inflammation, which are associated with disease such as Gaucher disease, Huntington’s disease, and other neurodegenerative disodres^21^.One study reported that an HDAC6 inhibitor modulated molecular chaperone proteins such as HSP90 and HSP70, influencing the folding of Peripheral Myelin Protein 22 (PMP22) protein in Charcot-Marie-Tooth disease. This modulation improved axonal integrity in nerves, alleviating clinical symptoms in an animal model^17^. Similar findings were reported in a study targeting Huntington’s disease, where the HDAC6 inhibitor was shown to enhance tubulin acetylation, stabilize microtubules, and improve axonal transport. In vivo, HDAC6i have been shown to alleviate behavioral deficits, restored synaptic function, reduced mutant huntingtin protein accumulation, and decreased inflammation and tau hyperphosphorylation^22^. Another domestic study investigated the potential of HADC6i as therapeutic agent for neuropathic Gaucher disease. This study demonstrated that HDAC 6 inhibitor reduced the expression of protein related to apoptosis and DNA damage, suggesting their therapeutic potential for this disease^19^. As a lysosomal storage disorder, neuropathic Gaucher disease highlights mechanisms that may be relevant to other inherited metabolic conditions, supporting further investigation of HDAC6 inhibition in diseases such as PKU. Some studies have demonstrated that HDAC6, through interactions with substrates such as α-tubulin, HSP90, and cortactin, regulates key cellular processes including gene transcription, DNA repair, and cell movement. These findings highlight the diverse biological functions of HDAC6 in maintaining protein quality control and cellular homeostasis. Research findings have shown that dysregulated HDAC6 expression or activity contribute to oncogenesis, suggesting HDAC6i may be a therapeutic approach^23,24^. Furthermore, preclinical results, including effects on PD-L1 regulation and reduced toxicity compared to combined HDAC and proteasome inhibitors, have yielded positive outcomes, emphasizing their potential in cancer therapy^25,26^.

Acetylated HSP90 enhances the stabilization of misfolded PAH proteins, preventing their degradation and partially restoring enzymatic activity. This effect was evidenced by the increased PAH expression observed in the liver of TSA-treated mice and is consistent with previous reports showing that HDAC6 inhibition enhances HSP90 acetylation, which in turn impairs its clearance function and allows stabilization of misfolded client proteins with residual activity^27^.

There have been no prior studies investigating the use of HDAC6i in the treatment of PKU. Notably, gene therapy using adenovirus vectors has shown potential in animal studies. Adeno-associated virus vectors (AAV) deliver functional PAH genes to liver cells, restoring enzymatic activity and normalizing phenylalanine metabolism. This approach offers the possibility of long-term correction of the metabolic defect, reducing the need for lifelong dietary restrictions^3,26^. A recent study demonstrated the therapeutic potential of the adeno-associated virus vector in *Pah*^*eun2*^ mice to effectively phenylalanine metabolism and pigmentation to achieve control phenylalanine levels with no significant adverse effects^28^. Despites of these outcomes, challenges such as immune responses to the vector, the durability of gene expression, and the high cost of therapy remain significant barriers to clinical application. Currently, a Phase 1/2 clinical trial (NCT04480567) is evaluating BMN-307, an AAV5-mediated gene therapy, in adults with PKU. This trial assesses the safety and efficacy of a single intravenous dose of the vector, with long-term follow-up through 2027^29^.

Traditional treatments, such as dietary therapy or BH_4_ supplementation, currently used in clinical practice, have limited efficacy and encounter compliance challenges. In contrast, HDAC6 inhibition directly targets intracellular protein misfolding, offering a potentially more sustainable and effective therapeutic option. HDAC6 uses key substrates such as HSP90 and α-tubulin, which are essential for maintaining structural stability and cellular homeostasis^20^. HDAC6i maintain acetylated HSP90, disrupting natural degradation pathways for misfolded proteins, including proteasomal degradation and autophagy^30^. This mechanism prevents the degradation of misfolded PAH proteins, facilitating their refolding and restoring partial function. Moreover, HDAC6i has the ability to modulate protein aggregation and cellular stress responses; this underscores their broader therapeutic potential in treating other metabolic and neurodegenerative diseases^31,32^.

Our study provides significant insights into advancing therapeutic development for PKU. First, this study is the first to investigate the therapeutic potential of HDAC6i in PKU, providing a new direction in the treatment of metabolic disorders. By targeting protein misfolding and degradation, HDAC6i offer a novel solution to overcome the challenges of existing treatments, such as limited efficacy and compliance issues with dietary therapy or BH_4_ supplementation. Second, it demonstrates therapeutic potential through both biochemical and phenotypic outcomes, as shown by reduced plasma phenylalanine levels and restored fur color in the PKU mouse model. Third, it highlights the molecular mechanism of HDAC6 inhibition, including enhanced acetylation of HSP90 and stabilization of mutant PAH proteins.

However, some limitations of the present study should be noted. First, this study was limited to a 12-week treatment period. Longer studies are needed to determine whether the reduction in phenylalanine levels can reach those of control group and to evaluate potential long-term adverse effects. Second, the use of only three mice per group restricts the statistical power and representativeness of the finding. Larger sample sizes will be necessary in future studies to obtain more reliable conclusion. Third, the high-dose group exhibited nephrotoxicity and mortality within eight weeks, emphasizing the need for further studies to optimize dosing regimens for improved safety and efficacy.

In this study, we have demonstrated that TSA effectively reduced plasma phenylalanine levels and ameliorated clinical symptoms in the *Pah*^*eun2*^ mouse model of PKU. Furthermore, this study allowed us to gain valuable insights into the biological function of HDAC6 and its potential as a therapeutic target for PKU and other disorders characterized by protein misfolding. Although HDAD6i is emerging as a potential therapeutic agent, its widespread clinical application has been constrained by problems including toxicity concerns. This study is expected to contributes to the development of novel therapeutic strategies for PKU.

## Methods

### Materials

TSA hydrocholoride, a potent inhibitor of HDAC6 was purchased from Santa Cruz Biotechnology (Santa Cruz, CA). The HDAC6i, TSA was dissolved in 0.74 mg/mL dimethyl sulfoxide (DMSO) and stored at -20 °C. The working concentration for this compound was 50 mg/kg. Phenylalanine Assay Kit was obtained from Abcam (Cambridge, UK). Additional materials included 10-kDa spin column (ab93349) purchased from Abcam, Microvette 500 blood collection tubes obtained from Orient Bio (Seongnam, South Korea), and 96-well black plates (Cat. No. 33396) purchased from SPL Life Sciences (Pocheon, South Korea).

### Animal study

All experimental protocols were review and approved by the Institutional Animal Care and Use Committee (IACUC) at Ewha Womans University (approval No. EWHA MEDIACUC 23 − 022). All animals were treated according to the Korea Food Drug Administration and National Institutes of Health guidelines for animal care. This study is reported in accordance with the ARRIVE guidelines (https://arriveguidelines.org) for animal research. The mice were provided free access to water and an 18% protein diet without phenylalanine restriction throughout the whole experimental period. At the end of the experimental period, mice were euthanized by exposure to carbon dioxide (CO₂) gas in a dedicated chamber, following institutional standard operating procedures to minimize suffering.

The experimental animal model consisted of PAH-deficient male mice *Pah*^*eun2*^ with the PAH mutation c.835 T > C (p.F263S) and wild-type BTBR male mice, aged 18–28 weeks at the start of the study. A pair of PAH-deficient mice, *Pah*^*enu2*^, were purchased (Jackson Laboratories, Bar Harbor, ME, USA) and maintained and bred to obtain sufficient numbers of mice for this study. This missense mutation changes the 263rd amino acid of the PAH protein from phenylalanine to serine, leading to hyperphenylalaninemia and phenotypic characteristics similar to human PKU. For genotyping, the homozygous c.835T > C mutation of the PAH gene in *Pah*^*enu2*^ mice was performed by Sanger sequencing, performed by Macrogen Inc. (Seoul, Korea), prior to the experiment. Subjects were divided into three groups, with three mice per group: a wild-type control group, a sham group administered DMSO, and an HDAC6i-treated group. Internal controls were included in the form of untreated wild-type mice (heterozygous) to ensure accurate comparisons and proper validation of experimental results. The treatment period lasted for 12 weeks, during which TSA (an HDAC6i) was administered intraperitoneally to the *Pah*^*eun2*^ mice at a dose of 50 mg/kg twice per week. Body weight was monitored twice weekly, along with observation of fur color changes. Venous blood sampling was performed using the facial vein of the mice. Blood was collected using Microvette 500 tubes, with 100–200 µL of blood obtained per sampling. To separate plasma from whole blood, the collected samples were subjected to centrifugation at 12,000 g for 5 min at 4 °C. The resulting plasma was carefully collected and stored at -70 °C. Serum phenylalanine concentrations were measured and compared every four weeks. An additional high-dose group, in which *Pah*^*eun2*^ mice were administered TSA at a dose of 100 mg/kg, was initially included in the experiment. However, these mice died at week 8, and necropsy findings indicated kidney enlargement and ascites accumulation, confirming dose-dependent toxicity. As a result, this group was excluded from the final analysis. At the end of the 12-week experiment, the mice were sacrificed, and tissue samples from the liver, kidney, brain, and heart were collected and preserved for further analysis. This procedure was performed for all groups, including wild-type control mice, HDAC6i-treated *Pah*^*eun2*^ mice, and untreated *Pah*^*eun2*^ mice.

## Plasma phenylalanine assay

Quantitative analysis of serum phenylalanine was carried out using phenylalanine assay kit (ab83376) from Abcam (Cambridge, UK). Serum samples were collected via venous blood sampling from each group at 0, 4, 8, and 12 weeks after administration. Whole blood was allowed to sit at room temperature for 1 h, followed by centrifugation at 12,000 rpm for 5 min at 4 °C to separate the serum. The isolated serum samples were stored at -70 °C until analysis. The samples were processed using a 10-kDa spin column (ab93349) to remove proteins and other interfering substances. A 96-well black plate was used for all measurements to optimize fluorescence detection and minimize background interference. Standard phenylalanine solutions were prepared according to the assay kit protocol to generate a standard curve. The plate was incubated at 37 °C for the specified duration as per the assay protocol to allow the enzymatic reaction to occur. Residual phenylalanine levels were measured using an ELISA reader (B229, Synergy) set to excitation/emission wavelengths of 535 nm/587 nm.

## Western blot

The mouse liver proteins were extracted using RIPA buffer (iNtRON biotechnology, Seongnam, South Korea) for 30 min on ice after centrifugation at 13,000 g for 5 min. For western blot analysis, 20 µg of total protein per sample was loaded in to 10% SDS-polyacrylamide gels. For the analysis of total proteins, membranes were blocked of 1 h with 5% skim milk (202100, BD difco) in 1% TBST. The antibodies used anti-acetylated HSP90 (600-401-981, Rockland Immunochemical, 1:500), anti-HSP90 (#4877, Cell Signaling, 1:1,000), and PAH (sc-271258, Santa Cruz, 1:2,000). Antibody against GAPDH (AM4300, Thermo, 1:10,000) was used as loading control. Immunoblotted membranes were developed with Clarity Western ECL Substrate (BIO-RAD) and imaged with Amersham™ ImageQuant™ 800 (Cytiva Life Sciences, formerly GE Healthcare Life Sciences, Marlborough, MA, USA). The images were analyzed using ImageQuant TL (version 10.0.261). Band intensities were quantified with ImageJ software (version 1.49). To ensure data transparency, the full-length uncropped blot images are presented in Supplementary Fig. S1.

### Statistical analysis

All tests were performed at least three times, and the results are reported as mean ± standard error of the mean (SEM). Statistical analyses were performed using Prism, version 10 (GraphPad Software, San Diego, CA, USA). One-way or Two-way ANOVA was used to determine significant differences among the three groups. Dunnett’s post hoc multiple comparisons test was used to compare the concentration of serum phenylalanine. A Tukey post hoc multiple comparisons test was used to compare the results of western blot analysis. *p* < 0.05 was considered significant.

### Euthanasia

Euthanasia was performed by gradually injecting carbon dioxide (DAEHAN SPECIAL GAS Co. LTD. Seoul, Korea) at a flow rate of 30–70% of the chamber volume per minute, and the mice were exposed for at least 1 min after their breathing stopped. The total exposure time was maintained for at least 5 min, and euthanasia was performed after confirming that the mice’s breathing and heartbeat had completely stopped. This euthanasia method is based on the American Veterinary Medical Association (AVMA) Guidelines for the Euthanasia of Animals (2020 edition).

## Supplementary Information

Below is the link to the electronic supplementary material.

Supplementary Material 1

## Data Availability

The raw Western blot images and quantification data generated during the current study have been deposited in Figshare and are publicly available at: [https://doi.org/10.6084/m9.figshare.28942610](https:/doi.org/10.6084/m9.figshare.28942610) . No new DNA or RNA sequencing data were generated in this study. The genotypic confirmation of the *Pahenu2* mouse model was conducted by Macrogen Inc. using Sanger sequencing for quality control purposes and is not part of the primary dataset analyzed in the study. All other relevant data are available from the corresponding author upon reasonable request.
