# Supplementary material for: Therapeutic effect of histone deacetylase 6 inhibitor for a mouse model of phenylketonuria
Source: Sci Rep. 2025 Nov 22;15:44998. doi: 10.1038/s41598-025-29143-7 (PMC12748783; doi:10.1038/s41598-025-29143-7)

Fig. S1: Full-length uncropped Western blot images related to the blot. Bands represent WT, Sham, and Treated groups.

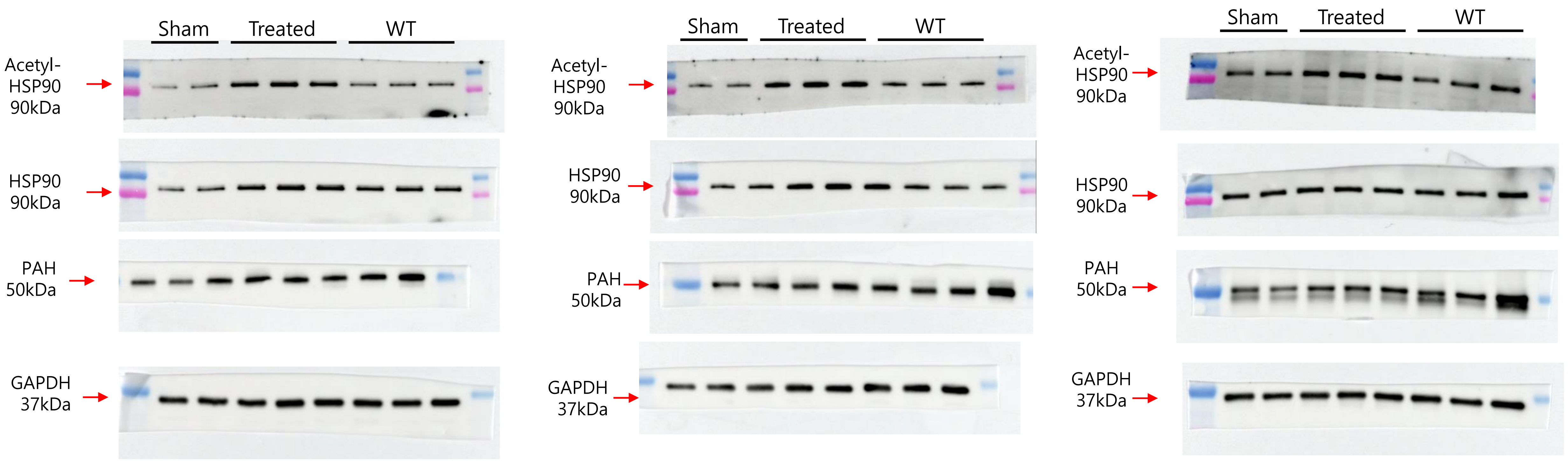

Supplement: Supplementary file 1 — Supplementary Material 1 [file 41598_2025_29143_MOESM1_ESM.pdf]
